# Novel assay to measure the plasmid mobilizing potential of mixed microbial communities

**DOI:** 10.3389/fmicb.2014.00730

**Published:** 2014-12-22

**Authors:** Uli Klümper, Ariadni Droumpali, Arnaud Dechesne, Barth F. Smets

**Affiliations:** Department of Environmental Engineering, Technical University of DenmarkKongens Lyngby, Denmark

**Keywords:** plasmid mobilization, permissiveness, RSF1010, RP4, plasmid transfer, conjugation, horizontal gene transfer

## Abstract

Mobilizable plasmids lack necessary genes for complete conjugation and are therefore non-self-transmissible. Instead, they rely on the conjugation system of conjugal plasmids to be horizontally transferred to new recipients. While community permissiveness, the fraction of a mixed microbial community that can receive self-transmissible conjugal plasmids, has been studied, the intrinsic ability of a community to mobilize plasmids that lack conjugation systems is unexplored. Here, we present a novel framework and experimental method to estimate the mobilization potential of mixed communities. We compare the transfer frequency of a mobilizable plasmid to that of a mobilizing and conjugal plasmid measured for a model strain and for the assayed community. With *Pseudomonas putida* carrying the *gfp*-tagged mobilizable IncQ plasmid RSF1010 as donor strain, we conducted solid surface mating experiments with either a *P. putida* strain carrying the mobilizing IncP-1α plasmid RP4 or a model bacterial community that was extracted from the inner walls of a domestic shower conduit. Additionally, we estimated the permissiveness of the same community for RP4 using *P. putida* as donor strain. The permissiveness of the model community for RP4 [at 1.16 × 10^-4^ transconjugants per recipient (T/R)] was similar to that previously measured for soil microbial communities. RSF1010 was mobilized by the model community at a frequency of 1.16 × 10^-5^ T/R, only one order of magnitude lower than its permissiveness to RP4. This mobilization frequency is unexpectedly high considering that (i) mobilization requires the presence of mobilizing conjugal plasmids within the permissive fraction of the recipients; (ii) in pure culture experiments with *P. putida* retromobilization of RSF1010 through RP4 only took place in approximately half of the donors receiving the conjugal plasmid in the first step. Further work is needed to establish how plasmid mobilization potential varies within and across microbial communities. This method has the potential to provide such insights; in addition it allows for the direct isolation of *in situ* mobilizing plasmids together with their endogenous hosts.

## INTRODUCTION

Plasmid transfer is believed to be a main mechanism in rapid bacterial adaption to environmental changes (Sørensen et al., 2005; Grohmann, 2011; Heuer and Smalla, 2012). Plasmids can be classified into two main groups based on the presence of genes associated with the transfer phenotype (Smillie et al., 2010). Conjugal plasmids encode a complete set of transfer genes needed to be self-transmissible. Mobilizable plasmids, on the other hand, lack some of the genes encoded in the transfer operon (*tra*), which encodes most of the functions involved in mating pair formation (MPF; Thomas and Nielsen, 2005).

**FIGURE 1:**
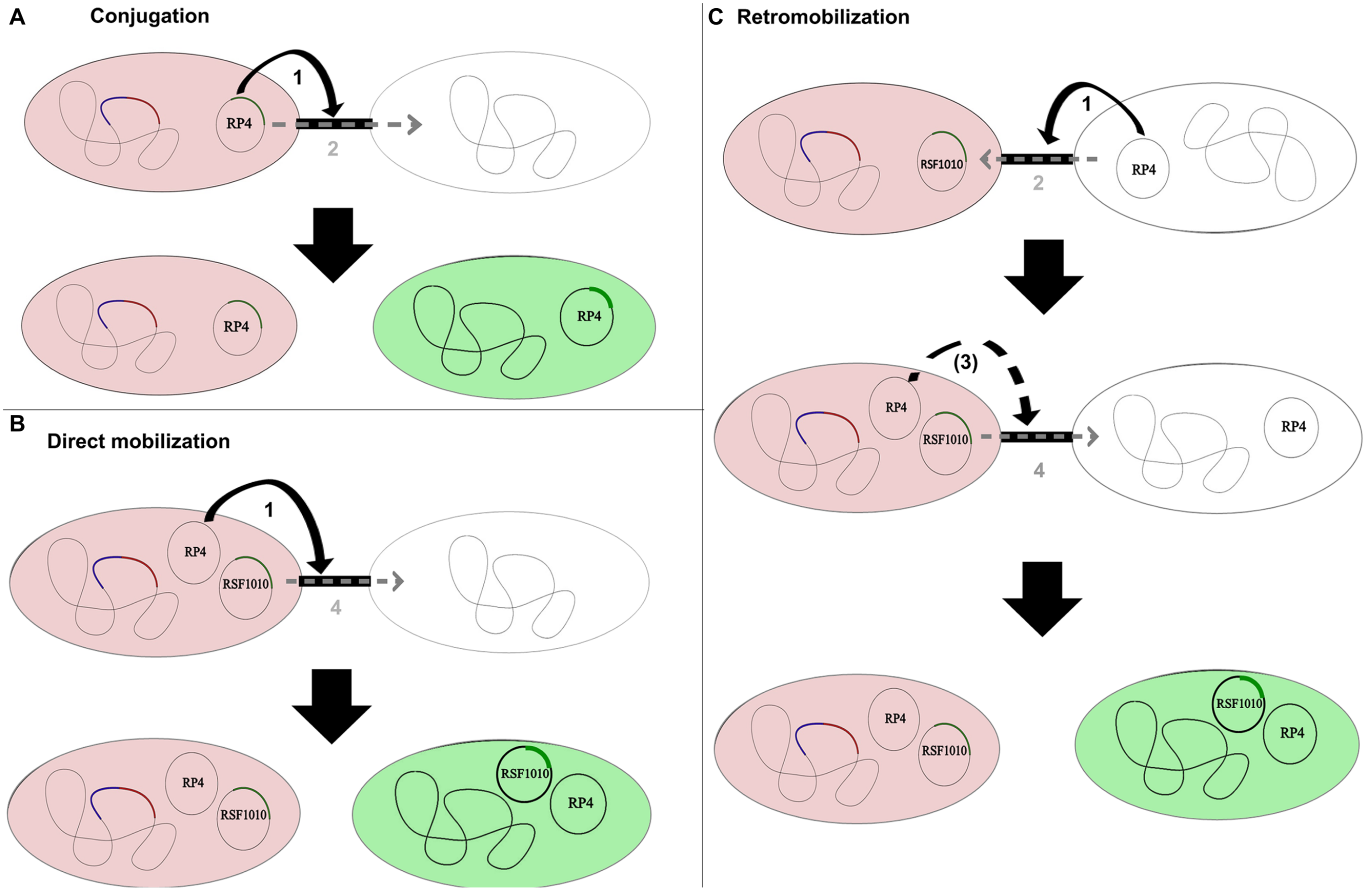
**Conjugation, direct mobilization, and retromobilization of the conjugative/mobilizable plasmid couple RP4/RSF1010.** In all shown combinations, the donor strains are displayed in red as chromosomally tagged with the red fluorescent protein gene *mCherry* and a *gfp* repressor gene (blue). Recipients transition from being colorless to being green after the *gfp*-tagged plasmid is transferred and thus freed from the chromosomal repression of the donor.** (A)** Conjugal transfer of the self-transmissible IncP-1 plasmid RP4. Step 1 illustrates the establishment of the pilus between donor and recipient as part of the type IV secretion system (T4SS) encoded by the conjugal plasmid. Step 2 displays the transfer of the conjugal plasmid through its own secretion system into the recipient. **(B)** Direct mobilization of the mobilizable IncQ plasmid RSF1010 from the donor to the recipient by the co-resident conjugal plasmid RP4. The conjugal plasmid establishes the pilus as part of its T4SS and interconnects donor and recipient strain (Step 1). The mobilizable plasmid does not encode for its own T4SS and transfers through the established pilus into the recipient cell (Step 4). The conjugal plasmid might or might not transfer along with the mobilizable plasmid in the direct mobilization process. **(C)** Retromobilization process of plasmid RSF1010, mobilized by a conjugal plasmid from the recipient cell. In this process, the conjugal plasmid from the recipient establishes the conjugal connection between recipient and donor (Step 1) and transfers from the recipient to the donor (Step 2). The mobilizable plasmid can subsequently transfer through the established connection (Step 4) or through a potential new connection established by the now co-resident conjugal plasmid (Step 3).

Conjugal plasmids possess an origin of transfer (*oriT*), a relaxase, type IV coupling proteins (T4CP) and a type IV secretion system (T4SS). The relaxase is a key protein of the conjugal machinery, common to all conjugal and mobilizable plasmids. Conjugal transfer of self-transmissible plasmids like the IncP-1α plasmid RP4 is based on pilus establishment between donor and recipient cells coded by the T4SS. The plasmid then transfers through the pilus into the recipient (**Figure 1**). Mobilizable plasmids encode only a *MOB* module (with or without the T4CP) and need the MPF apparatus of a co-resident (i.e., located within the same cell) conjugal plasmid to be transmissible by conjugation (Smillie et al., 2010). To be transferred, they take advantage of a conjugal plasmid that initiates replication through expression of its *rep* genes. These genes are involved in pilus formation and connection of the relaxosome with proteins enabling passage of the DNA across the membranes (Yano et al., 2013). Direct mobilization involves a presently co-resident conjugal plasmid; in retromobilization the donor cells (harboring the mobilizable plasmid) must first receive a mobilizing conjugal plasmid from the recipient, which thereafter mobilizes the mobilizable plasmid toward the recipient (**Figure 1**).Therefore, microbial communities need a high intrinsic conjugal plasmid content to allow mobilization of mobilizable plasmids with potentially useful genetic content, when no co-resident conjugal plasmids are present in the newly introduced donor strain.

The most well-studied non-self-transmissible, mobilizable plasmids belong to the IncQ group. Compared to the broad host range IncP-1 conjugal plasmids, they are relatively small (5.1–14.2 kb; Loftie-Eaton and Rawlings, 2012). Thanks to their host independent replication system, these plasmids have a broader host range than any other known replicating components in bacteria (Meyer, 2009). They can be conjugally mobilized by a variety of different plasmid encoded type IV transporters (Meyer, 2009) as well as through integrative and conjugative elements (ICEs; Lee et al., 2012), both often at high frequencies (Gregory et al., 2008; Meyer, 2009).

Mobilization by the IncP-1 plasmids has contributed extensively to the dissemination of IncQ plasmids (Meyer, 2009) and the coupling of the transfer machinery of the IncP-1 RP4 plasmid to mobilize the IncQ RSF1010 plasmid has been well studied (Lessl et al., 1993; Haase et al., 1995).

In order to assess a conjugal plasmid’s potential contribution to horizontal gene transfer in a microbial community, the permissiveness of the community toward the plasmid is a main parameter. We have defined permissiveness as the fraction of a community able to receive and maintain a target exogenous plasmid (Musovic et al., 2010; Klümper et al., 2014). Different factors such as phylogenetic diversity, cell density, and various environmental stress factors may affect community permissiveness (Musovic et al., 2010; Heuer et al., 2011). While some bacteria are known to exude signal molecules in order to obtain plasmids (Hirt, 2002), permissiveness toward a self-transmissible, conjugal plasmid is probably a passive trait of the bacterial community. The ability of a community to receive genes located on mobilizable non-self-transmissible plasmids, on the other hand, would rely on the community’s own content of conjugal plasmids. While the spread and contribution of conjugal plasmids to gene exchange has been intensely studied (Heuer et al., 2012; Shintani et al., 2014; Zhang et al., 2014), the mobilization potential of microbial communities and the contribution of mobilizable plasmids to horizontal gene flow have been comparably poorly studied (Top et al., 1995). Exogenous isolation techniques to capture mobilizing and mobilizable plasmids from natural communities have been developed (Top et al., 1994; van Elsas et al., 1998; Smalla et al., 2000). However, the characterization of communities based on their mobilization potential has mainly been carried out using indirect measures through triparental matings where both donor and terminal recipient were artificially introduced to the communities (Hill et al., 1992). For example, manure addition was shown to increase a soil microbial community’s ability to support mobilization of a mobilizable plasmid between two introduced strains through an increased intrinsic plasmid content (Götz and Smalla, 1997). Direct mobilization of mobilizable plasmids into indigenous bacteria of a mixed community has been detected before (Hill et al., 1992; van Elsas et al., 1998), but was never directly quantified.

Here, we present a novel experimental method to estimate the plasmid mobilization potential of a mixed bacterial community, using IncQ RSF1010 as model plasmid. We quantify the mobilization potential of a model community extracted from a domestic shower conduit. We evaluated the transfer frequency by comparing it to the community’s permissiveness toward the mobilizing, conjugal plasmid RP4. We finally related the observed transfer frequencies to those measured in transfer between isogenic strains. We additionally aimed to isolate transconjugants that mobilized the RSF1010 plasmid, assuming that retromobilization is the main mobilization process.

## MATERIAL AND METHODS

### PRINCIPLE OF PLASMID TRANSFER DETECTION

**FIGURE 2:**
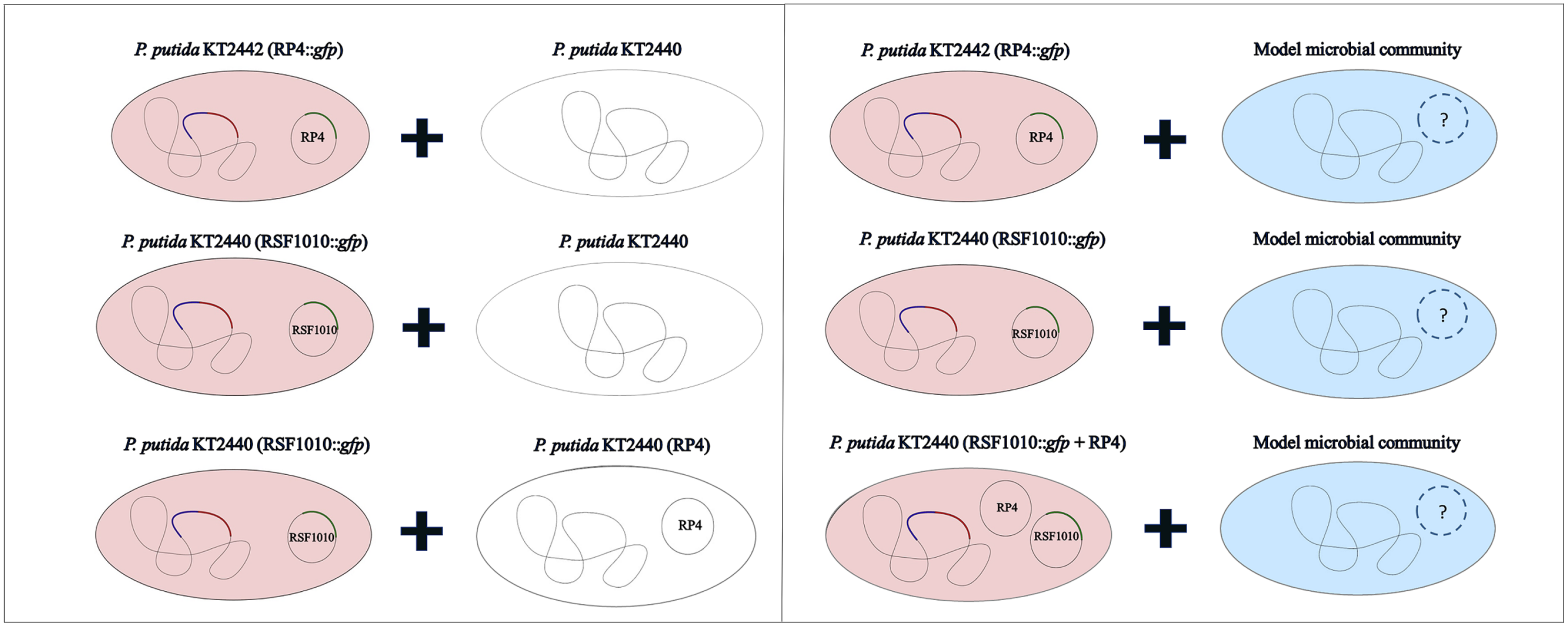
**Overview of executed filter mating combinations**.

The recipient community was challenged with various plasmid combinations introduced through *Pseudomonas putida* in solid surface filter matings (**Figure 2**). All strains used or constructed for this study can be found in **Table 1**. The plasmids (**Table 2**) were marked with a genetic tag encoding a conditionally expressible fluorescent marker. The used entranceposon (Bahl et al., 2009) carries a *lacI^q^* repressible promoter upstream of the *gfpmut3* gene, coding for the green fluorescent protein (*gfp*). The plasmid donor strain was chromosomally tagged with a gene cassette encoding constitutive red fluorescence and constitutive *lacI^q^* production. As a result, there is no *gfp* expression in the donor strain, but upon plasmid transfer to recipient bacteria, *gfp* expression is possible, resulting in green fluorescent cells or microcolonies, which can be detected and quantified by fluorescence microscopy or sorted by fluorescent activated cell sorting (FACS), respectively. *P. putida* KT2440 served as the donor strain in all the experiments, and was tagged through electroporation with plasmid pGRG36-*lacI^q^-Km^R^-Lpp-mCherry* carrying both the transposase genes and the Tn7 *lacI^q^-Lpp-mCherry-Km^R^* region for specific integration of the *lacI^q^-Lpp-mCherry-Km^R^* gene cassette into the chromosomal attTn7 site as described earlier (Bahl et al., 2009).

**Table 1:** Donor and recipient strains used in this study.

Species/strain	Plasmid	Resistance (μg/mL)	Chromosomal markers	Reference
*Pseudomonas putida* KT2442	RP4::*gfp*	*Km^R^, Amp^R^, Tet^R^* (50, 100, 10)	*Rif^R^*	Musovic et al. (2010)
*P. putida* KT2440	RSF1010::*gfp*	*Strep^R^* (100)	*lacI^q^-pLpp-mCherry, Km^R^*	This study
*P. putida* KT2440	RSF1010::*gfp*, RP4	*Strep^R^, Amp^R^, Tet^R^, Km^R^* (100, 100, 40, 50)	*lacI^q^-pLpp-mCherry, Km^R^*	This study
*P. putida* KT2440	*–*	*–*	*–*	Nelson et al. (2002)
*P. putida* KT2440	RP4	*Tet^R^, Km^R^, Amp^R^* (40, 50, 100)	*–*	This study

**Table 2:** Plasmids used in this study.

Plasmid	Transfer	Size	Incompatibility	Resistance (μg/mL)	Host range	Degradation pathways	Reference
RP4	Conjugal	60 kb	IncP-1α	*Amp^R^, Km^R^, Tet^R^* (100, 50, 20)	broad	BP, 4CBP	Barth and Grinter (1977)
RSF1010	Mobilizable	8.7 kb	IncQ	*Strep^R^* (100)	broad	Arginine, Ornithine	Honda et al. (1991)

The 8.7 kbp IncQ plasmid, RSF1010, originally isolated from *Escherichia coli* (Scholz et al., 1989), harbors streptomycin and sulphonamide resistance determinants and genes for the degradation of arginine and ornithine. For *gfp*-tagging the *P*_A10403_-*gfpmut3-Km^R^* section of entranceposon [*Km^R^*, *P_A10403_-gfpmut3*] was amplified by PCR, subjected to subsequent enzyme digestion and ligated to the RSF1010 vector cut with the same enzyme. The correct insert location at the enzyme cut site of [*Km^R^*, *P*_A10403_-*gfpmut3*] in plasmid RSF1010 was confirmed by sequencing from the inserted fragment in one direction using primer Seq_Bw_Ent_gfp: 5′-GCCAGAACCGTTATGATGTCGG-3′. The selected *gfpmut3*-tagged RSF1010 (abbreviated as RSF1010*::gfp*) plasmid was finally introduced by transformation into the donor strain, *P. putida* KT2440::*Km^R^-Lpp-mCherry*.

A donor *P. putida* KT2440::*Km^R^-Lpp-mCherry* with both RSF1010::*gfp* and the wild type conjugal plasmid RP4 was also constructed. The previously created donor strain *P. putida* KT2440::*Km^R^-Lpp-mCherry* carrying the RSF1010::*gfp* plasmid was mated with *E. coli* J5 harboring an untagged version of the RP4. Mating was carried out on microfiber filters (GF/C Whatman filter, 24 mm). Cells were detached from the mating filters and *P. putida* donor strains hosting both plasmids were selected for on 10 mM citrate medium supplemented with streptomycin and tetracycline and checked for red and green fluorescence after IPTG induction of *gfp*.

### DONOR AND RECIPIENT STRAIN GROWTH AND PREPARATION

The *P. putida* recipient and donor strains were grown overnight on R2A medium supplemented with the plasmid specific antibiotics (**Table 2**) and harvested by centrifugation at 10,000 × *g* for 10 min. Harvested cells were resuspended and washed twice with sterile 0.9% NaCl solution to remove residual antibiotics and thereafter adjusted to a bacterial density of 3 × 10^6^ bacteria/mL using Thoma chamber counts and sterile 0.9% NaCl solution for dilutions.

### RECIPIENT COMMUNITY EXTRACTION AND PREPARATION

As model recipient microbial communities, we extracted biofilms that colonized the inner walls of a domestic shower PVC hose from a private residence. The shower hose was first drained in a sterile 50 mL Falcon tube. The emptied hose was then incised with a sterilized steel scalpel blade and the biofilm at its inner surface removed by scraping. The removed biofilm was transferred to the same 50 mL Falcon tube. The suspension was centrifuged for 8 min at 10.000 × *g*. The pellet was resuspended in 5 mL TTSP [tetrasodium pyrophosphate (50 mM), Tween 80^®^ (0.05%)], vortexed at maximum speed for 5 min, and sonicated 60 s in a Branson Sonifier 250 (Branson, MO, USA) at 40% power at 200 W to disrupt cell aggregates. The bacterial suspension was then filtered through a sterile 20 μm pore-size filter. This filtrate was used as the recipient community in mating assays after adjusting the bacterial density to ∼3 × 10^6^ bacteria/mL, as confirmed by Thoma chamber counts.

### SOLID SURFACE FILTER MATING ASSAY

The recipient communities were challenged with the plasmids introduced through the constructed donor via solid surface filter matings (Musovic et al., 2010) at a 1:1 initial donor to recipient cell ratio and an initial density of approximately 30,000 bacteria/mm^2^ filter surface area, with 10-fold diluted R2A as solid 1.5% agar mating medium. Conjugation was verified by epifluorescence stereomicroscopy after 48 h incubation at room temperature and the transfer events quantified (Musovic et al., 2010). R2A was chosen as filter mating medium as it is presumed optimal for water borne organisms (Reasoner et al., 1979). However, to simulate low nutrient conditions typical of drinking water distribution systems (Boe-Hansen et al., 2002), the R2A medium was diluted to the maximum extent possible, while maintaining high enough bacterial activity for growth of microcolonies, to establish donor to recipient cell contact during the mating, and for expression of the plasmid encoded *gfp*-gene after plasmid transfer. Five different dilutions of R2A (1:5, 1:10, 1:50, 1:100, 1:1000) were tested and the 10-fold diluted R2A was finally chosen, as it was the highest dilution at which transconjugants were still observed for all tested plasmids.

### VISUALIZATION AND QUANTIFICATION OF TRANSFER EVENTS BY STEREOMICROSCOPY AND IMAGE ANALYSIS

Successful plasmid transfer was visualized *in situ* by stereomicroscopy and quantified by automated image analysis (Image Pro Plus 7.1; Media Cybernetics, Silver Spring, MD, USA) as previously described (Musovic et al., 2010), using a Leica MZ16 FA fluorescence stereomicroscope equipped with a 10x plan apochromatic objective, a 10× eyepiece (10×/21B), a 40× magnification zoom. Conditions for *gfp*- and *mCherry*-based fluorescence detection were 480/20 nm with emission at 525/40 nm and 580/25 with emission at 650/60 nm, respectively, and images were acquired with a Leica DFC300 fluorescence camera. A representative scanning zone of 7 × 7 fields of 980 × 732 μm each were analyzed per filter. With a total filter area of 270 mm^2^, the scanned and quantified area corresponded to approximately 13% of the total filter area. Triplicate filters were analyzed for each donor/recipient combination.

**FIGURE 3:**
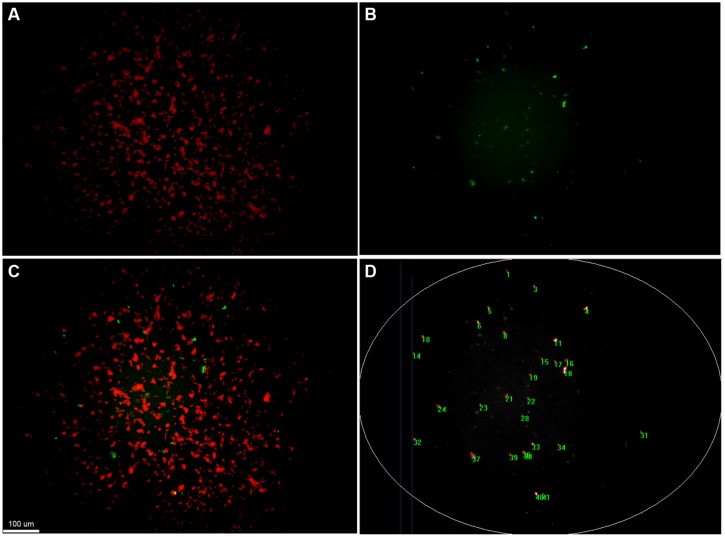
**Detection and quantification of transconjugant microcolonies by fluorescent microscopy.** Fluorescence based stereomicroscopic images and image analysis of an example filter mating of *Pseudomonas putida* KT2440::*lacI^q^-Lpp-mCherry-Km^R^* (RSF1010::*gfp*) with the recipient community. **(A)** corresponds to the red fluorescent channel, displaying donor microcolonies. **(B)** shows the green fluorescent channel, corresponding to the transconjugal microcolonies that received the plasmid through retromobilization. **(C)** is a composite image of both channels with increased contrasts. Transconjugal microcolonies can be found in direct proximity to donor colonies. **(D)** illustrates counting of transconjugal colonies through a macro that increases contrast of the images, subtracts background, eliminates the poorly illuminated corners and counts green fluorescent object larger than 4 μm^2^.

Quantification of transfer events was performed with a custom-made macro written in Image Pro Plus 7.1. This macro successively extracts and subtracts the background from the original image, performs a best-fit equalization of the image intensity, before detecting bright objects larger than 4 μm^2^ based on automatic segmentation. Analysis of images was limited to the brightly illuminated elliptic central area of the field of view (**Figure 3**). All images were manually controlled for enumeration errors, and values corrected if deviations were noted. The number of *gfp*-positive colonies (transfer events) detected was scaled up to the total filter area and transfer frequency was calculated by dividing this number by the number of potential recipients originally placed on the filter.

### CELL COLLECTION AND FLUORESCENCE ACTIVATED CELL SORTING OF TRANSCONJUGANTS

Cells from the filter mating between *P. putida* (RSF1010::*gfp*) and the model community were removed by vortexing in 2 mL of a 0.9% NaCl-solution for 3 min. Flow cytometric detection of cells and *gfp-*based isolation of transconjugants were carried out using a FACSAria IIIu Flowcytometer (Becton Dickinson Biosciences, San Jose, CA, USA), as previously described (Klümper et al., 2014).

## RESULTS

### PERMISSIVENESS OF THE RECIPIENT COMMUNITY FOR CONJUGAL IncP-1 PLASMID RP4

**FIGURE 4:**
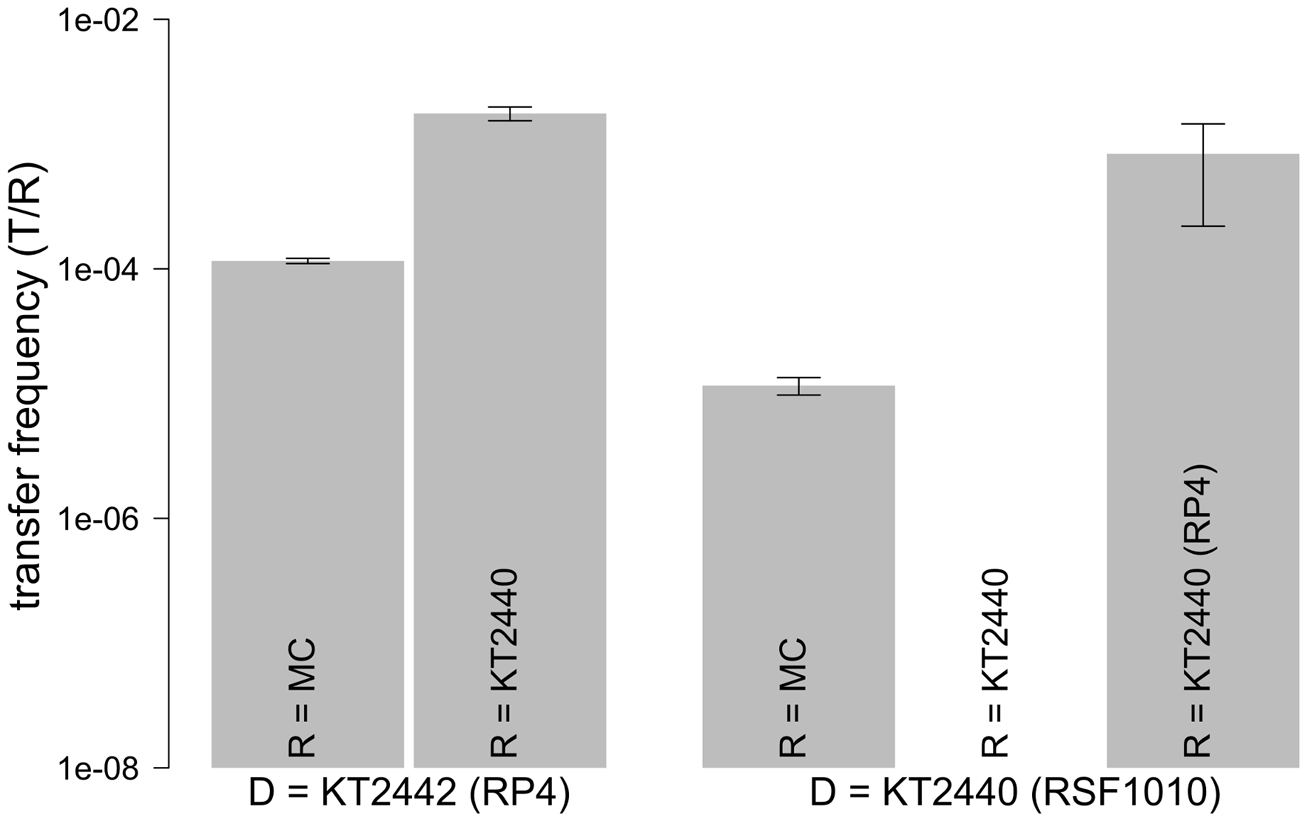
**Transfer frequencies of RSF1010 and RP4.** Transfer frequencies defined as transconjugant microcolonies per recipient were obtained in solid surface filter matings with the recipient community and in *P. putida* intrastrain matings. Values are shown as mean of triplicates with standard error of mean. The *gfp*-tagged plasmid in the donor strain (D) is shown on the x-axis. RP4 or RSF1010 were each introduced through KT2440 or KT2442 (*P. putida* KT2440/KT2442::*lacI^q^-Lpp-mCherry-Km^R^*) into the recipients. Recipients (R) are shown within the bars (MC: model community; KT2440: *P. putida* KT2440). For the combination D= KT2440 (RSF1010) with R= KT2440 no transfer was observed.

We explored the intrinsic ability of an extracted model microbial community to mobilize the broad host range mobilizable plasmid RSF1010 as well as its ability to receive the conjugal broad host range plasmid RP4. Both plasmids were introduced via a red fluorescent-tagged donor *P. putida* in which plasmid encoded *gfp* expression is repressed (**Table 1**). Microscopic examination and enumeration of the mating events (**Figure 3**) between the recipient microbial community and *P. putida* (RP4::*gfp*) revealed a transfer frequency of 1.16 × 10^-4^ transconjugants per potential recipient (T/R; **Figure 4**). A higher transfer frequency (1.76 × 10^-3^ T/R) was observed in the mating assay using isogenic *P. putida* donor and recipient strains (**Figure 4**). In this experiment, all recipients were obviously within the plasmid host range and any incompatibility effect with RP4 could be ruled out because they were all initially plasmid-free. Hence, the observed transfer frequency in these intrastrain experiments was not limited by the recipient permissiveness, but only by donor promiscuity (the fraction of donor cells expressing conjugal genes), successful completion of initiated plasmid transfer events to *P. putida* recipient cells, and the degree of donor–recipient contact saturation.

We can now express the community’s permissiveness against the defined co-culture experiments: The community permissiveness for the conjugal RP4 (1.16 × 10^-4^ T/R) is divided by the conjugal transfer frequency of plasmid RP4 in intrastrain matings, where all *P. putida* recipients can potentially take up RP4 (1.76 × 10^-3^ T/R), as a standard. The resulting community permissiveness for RP4 is 0.066 RP4 intrastrain equivalents.

### MOBILIZING POTENTIAL OF THE RECIPIENT COMMUNITY FOR PLASMID RSF1010

When the model community was challenged with *P. putida* (RSF1010::*gfp*), a transfer frequency of 1.16 × 10^-5^ T/R was measured. This value is one order of magnitude lower than the community’s measured permissiveness for the conjugal plasmid RP4 (**Figure 4**).

In these experiments RSF1010 must have been retromobilized into the recipient community by cells carrying IncQ compatible mobilizing conjugal plasmids (**Figure 1**). In order to explore the retrotransfer frequency of RSF1010 further, isogenic *P. putida* strains were used to execute two different intrastrain matings, taking advantage of all *P. putida* recipient cells being potential RSF1010 hosts. In the first experiment, a plasmid-free, non-*mCherry*-tagged *P. putida* strain served as recipient. In the second experiment, a non-*mCherry*-tagged *P. putida* strain hosting the untagged wild-type conjugal, mobilizing RP4 plasmid served as recipient. In the first experiment no RSF1010 transfer was observed, consistent with RSF1010’s non-self-transmissible nature. In the second experiment with *P. putida* (RP4) as recipient, retrotransfer was observed, with a measured frequency of 8.34 × 10^-4^ T/R. Successful RSF1010 retrotransfer requires initial conjugal plasmid transfer from recipients to RSF1010 donors, before RSF1010 is mobilized and retransferred to the recipients (Top et al., 1992; **Figure 1C**).

RSF1010 retrotransfer frequency by *P. putida* (RP4) results from a combination of the RP4 transfer process from the recipient to the donor (**Figure 1C** Steps 1 and 2) and the subsequent mobilization of RSF1010 through the now co-resident RP4 plasmid (**Figure 1C** Steps 3 and 4). It can be contrasted with the measured RP4 intraspecies transfer frequency of 1.76 × 10^-3^ T/R. RP4 intrastrain transfer corresponds to the first two steps in RSF1010 retrotransfer (**Figure 1A**). Hence, the probability for a cell that recently acquired RP4 via conjugal transfer to mobilize RSF1010 can be estimated at 47.4% [8.34 × 10^-4^ T/R for *P. putida* (RSF1010::*gfp*) to *P. putida* (RP4) divided by 1.76 × 10^-3^ (T/R) for *P. putida* (RP4::*gfp*) to *P. putida*]. For this specific pair of mobilizing and mobilizable plasmid, retrotransfer is high (**Figure 4**).

The retrotransfer of RSF1010 to the recipient community occurs at a frequency of 10% compared to its permissiveness for the RP4 plasmid. Still, as shown above, mobilization of RSF1010 is realized only approximately every second time a conjugal plasmid is transferred from the recipient community into the donor strain, based on mobilization through RP4. If all these potential mobilization events were realized, the maximal mobilization potential of the recipient community is reached. The theoretical maximal mobilization potential toward RSF1010 can be quantitatively assessed as 2.45 × 10^-5^ T/R by dividing its transfer frequency toward the community (1.16 × 10^-5^ T/R) by the now established 47.4% probability of retrotransfer. When subsequently dividing 2.45 × 10^-5^ T/R through the community’s permissiveness toward RP4 (1.16 × 10^-4^ T/R) as a standard, this results in 0.211 RP4 permissiveness equivalents as the maximal mobilization potential.

### POTENTIAL COMMUNITY PERMISSIVENESS TOWARD MOBILIZABLE PLASMID RSF1010

In a final experiment, we quantified the intrinsic permissiveness of the model community for RSF1010. To do so, we augmented the community’s own RSF1010 mobilizing potential by adding an exogenous RSF1010 mobilizing strain. Hence, the recipient community was challenged with *P. putida* hosting both the RSF1010::*gfp* and the wild-type RP4, which can directly mobilize RSF1010 (**Figure 1B**). The observed transfer frequency of RSF1010 in this mating was 3.14 × 10^-3^ T/R. This frequency is, surprisingly, higher (∼30-fold) than the community’s permissiveness for RP4. As expected, this value is also substantially higher (∼2 orders of magnitude) than the RSF1010 mobilization frequency (**Figure 4**) relying on the community’s inherent retromobilization potential only.

### FACS BASED SORTING OF RSF1010 TRANSCONJUGANTS

Cell suspensions from matings between the recipient community and *P. putida* (RSF1010::*gfp*) were collected, resuspended and subjected to FACS to isolate green fluorescent transconjugants (Klümper et al., 2014). 200 transconjugants were successfully sorted, despite a sorting time exceeding 24 h, due to the low initial relative abundance of transconjugant cells at less than 1:1,000,000 events sorted.

## DISCUSSION

Plasmids of the promiscuous, conjugal IncP-1 group illustrate the enormous potential of horizontal gene transfer among an extremely wide variety of gram-negative and gram-positive bacterial species (Gelder et al., 2005; Klümper et al., 2014; Musovic et al., 2014; Shintani et al., 2014). Studies on conjugal gene flow mainly focused on the passive characteristics of a mixed community to receive self-transmissible plasmids. Former approaches to assess the mobilization potential of mixed communities were using an indirect approach through triparental matings where both donor and terminal recipient were artificially introduced to the communities (Hill et al., 1992; Götz and Smalla, 1997) and even capture the mobilizing (van Elsas et al., 1998) or mobilizable (Smalla et al., 2000) genetic elements from natural communities. This study is the first one to directly quantify the potential of a microbial community to actively mobilize non-self-transmissible, mobilizable plasmids to its indigenous bacteria. It also illustrates how the community’s intrinsic plasmid content can contribute to an increased gene uptake potential. To estimate the maximum mobilization potential of a community, we utilized filter matings at maximized cell-to-cell contact of donor and potential recipients (Musovic et al., 2010). The spatial limitations for contact in water distribution systems might be small compared to other environments like the ones reported for soil (Dechesne et al., 2005). However, the initial invasion of the plasmid donor into the biofilm community might be limited to the surface of the biofilm and further reduced at high water flow conditions (Licht et al., 1999; Merkey et al., 2011; Król et al., 2013). Therefore, using our maximum cell-to-cell contact assay instead of natural conditions allows every single recipient cell to establish contact with donor cells and potentially engage in gene transfer. However, using this assay might limit the retransfer potential of the plasmid from new transconjugants to further recipients. Recipients that newly acquired the plasmid might only be surrounded by *P. putida* donor cells and not by other cells from the recipient community and can thus not retransfer the plasmid to other recipients. This retransfer process can especially be crucial for mobilizable plasmids. The first retromobilization transfer event leads to the co-occurrence of the mobilizable plasmid with the mobilizing conjugal plasmid(s) in the same cell. Through this co-occurrence the transconjugant cell significantly increases its transfer frequency of the mobilizable plasmid to the recipient community by switching the mechanism from retromobilization to direct mobilization, thereby omitting the steps involved in transferring the mobilizer to the donor cell. We measured a more than 300-fold increase in plasmid transfer for *P. putida* to the mixed community between retro- to direct mobilization. This large increase in transfer frequency was also reported earlier with a difference of over three orders of magnitude for direct mobilization versus retromobilization for a different mobilizable plasmid among pure strains (Top et al., 1995). Therefore, experiments that assess how this retransfer process influences the mitigation and invasion of a mobilizable plasmid from the initial donor through a mixed and spatially stratified biofilm community might be needed. To conclude, once mobilizable plasmids are in co-occurrence with a promiscuous mobilizing plasmid, they can significantly contribute to horizontal gene transfer in mixed communities.

We show here that the IncQ model plasmid RSF1010 can be easily mobilized by the bacterial community extracted from a household water distribution system. The permissiveness of this microbial community toward the conjugal plasmid RP4 is comparable in magnitude with that measured in diverse soil communities (Musovic et al., 2014). The lower permissiveness toward RP4 measured for mixed recipient communities compared to *P. putida* intraspecies transfer results primarily from the inability of a fraction of the bacterial community to either receive, transiently maintain, or express plasmid encoded genes.

The community’s potential to retromobilize and subsequently receive RSF1010 is only one order of magnitude lower than its permissiveness toward RP4. This surprisingly high transfer frequency may result from the fact that IncQ plasmids have a broader host range than any other known replicating component in bacteria (Meyer, 2009) combined with an extremely efficient transfer mechanism (Gregory et al., 2008; Meyer, 2009). The numbers appear even higher taking into account that in pure culture experiments with *P. putida*, only half of the microcolonies that recently received RP4 retromobilized RSF1010. Earlier retrotransfer experiments between two *E. coli* strains (Top et al., 1992) showed T/R ratios within the same orders of magnitude (10^-3^–10^-4^) as our intrastrain matings. But, they suggested that retrotransfer of the mobilizable plasmid appears at rates lower than 1% once the first step of acquiring a conjugal plasmid is realized. In that work transfer was quantified based on single cells and after 2.33 h. Our far higher numbers (∼50%) might therefore result from quantifying transfer on a microcolony basis after 48 h. Only one retrotransfer event within a microcolony is needed for quantification as successful transfer event and due to increased incubation time retrotransfer can happen not only through the initial, but also through newly established conjugal pili. Nonetheless, the observed retromobilization requires the presence of mobilizing, conjugal plasmids within the permissive fraction of the recipients. Other mobilization possibilities involve conjugation-independent transfer of plasmids through the formation of nanotubes from members of the complex community toward the donor cells (Dubey and Ben-Yehuda, 2011), but are only realized if nanotubes from the recipient to the *P. putida* donor are established. Therefore, a high intrinsic conjugal plasmid content of the model recipient community in combination with RSF1010’s efficient transfer mechanism is the most likely reason for the observed high mobilization potential.

IncP type IV secretion systems can conjugally connect a large variety of organisms (Grahn et al., 2000; Thomas and Nielsen, 2005; Klümper et al., 2014). But like the plasmids encoding them, they are evolutionary adapted to connect their mainly Gram-negative hosts. These self-transmissible plasmids might easily reach dead ends after being transferred, if the secretion system is not encoded efficiently for retransfer in the new host. Contrarily, mobilizable plasmids might less frequently reach dead ends once acquired, since they can utilize the conjugal connections build through adapted resident plasmids in their new host (Meyer, 2009) or through ICEs (Lee et al., 2012). Additionally, mobilizable plasmids are relatively stable, as their high copy number (Meyer, 2009) increases retention in a host until new transfer becomes possible. These two facts in connection with their strictly host-independent initiation of replication helps them to sustain in a very broad host range, including *Pseudomonas sp*., related species in the Proteobacteria, as well as phylogenetically distant species within the Firmicutes, Actinomycetes and even Cyanobacteria (Meyer, 2009) or plants (Buchanan-Wollaston et al., 1987). Consequently, RSF1010, as a mobilizable plasmid, has a far higher replication host range than RP4. RSF1010 can even spread to a mixed community at a more than 30-fold higher transfer frequency when directly mobilized through co-occurring plasmid RP4 in the same donor cell compared to RP4 itself. Therefore, mobilizable plasmids might contribute to long term gene spread and acquisition to a so far underestimated extent, especially in environments with high intrinsic mobilizing plasmid content. In our current experiment, we use a simplified system and are able to deliver insights into the mobilization potential of a community at the first acquisition event of a newly introduced mobilizable plasmid. The wide variety of mobilization systems possibly involved might not resemble the one encoded by RP4 in efficiency. Still, equivalents based on the community’s permissiveness toward RP4 can be used here, since long term maintenance and retransfer are not taken into account. For more complex natural systems and experiments that allow extensive retransfer we recommend assessing the intrinsic mobilization potential of microbial communities based on absolute transfer frequencies, as the transfer and maintenance processes of RSF1010 and RP4 differ too much in the long term.

Apart from quantification of the mobilization potential, the method presented here provides several possibilities to study plasmid ecology and mobilization mechanisms. FACS based sorting of RSF1010 carrying transconjugants from the recipient community was possible. Studying the diversity of transconjugants might provide insights into the enormous host range of mobilizable plasmids, compared to those of broad host range conjugal plasmids (Klümper et al., 2014). But the high amount of sorting time prohibits intensive studies at this point. However, taking advantage of FACS sorting, even at low speed, new possibilities for plasmid isolation emerge. The mobilizing, conjugal plasmid can, now, after retromobilization, co-occurring with RSF1010 in the transconjugant, be subsequently isolated within its original environmental host. Compared to common exogenous plasmid isolation techniques our method has the potential to also capture plasmids that are only transiently hosted and therefore quickly lost in the introduced capturing strains. Since these plasmids remain stable in their original hosts, we gain the ability to isolate them with our method. Isolated plasmids need therefore only stable maintenance in their natural hosts rather than in an artificially introduced strain. This increases the range of obtainable plasmids and immediately supplies information on where they naturally occur. This method reverses the exogenous isolation technique for mobilizable plasmids (Top et al., 1994) and is cultivation independent. Additionally using the tools presented here in combination with FACS sorting, single cell observations to better understand the exact mechanisms proposed for retromobilization (Top et al., 1992, 1995) might become possible.

In conclusion, this method is the first one to assess the plasmid mobilization potential of a microbial community on a quantitative level by estimating transfer frequencies through fluorescent microscopy. Using the new method, we discovered that a mixed microbial community has the potential to easily mobilize a newly introduced mobilizable plasmid at high rates compared to a conjugal plasmid. We also showed that the mobilizable plasmid is spread at far increased frequencies once directly mobilized by a co-occurring conjugal plasmid from within the same cell.

## Conflict of Interest Statement

The authors declare that the research was conducted in the absence of any commercial or financial relationships that could be construed as a potential conflict of interest.
